# Finite-size scaling of O(*n*) systems at the upper critical dimensionality

**DOI:** 10.1093/nsr/nwaa212

**Published:** 2020-08-31

**Authors:** Jian-Ping Lv, Wanwan Xu, Yanan Sun, Kun Chen, Youjin Deng

**Affiliations:** Department of Physics, Anhui Key Laboratory of Optoelectric Materials Science and Technology, Key Laboratory of Functional Molecular Solids, Ministry of Education, Anhui Normal University, Wuhu 241000, China; Department of Physics, Anhui Key Laboratory of Optoelectric Materials Science and Technology, Key Laboratory of Functional Molecular Solids, Ministry of Education, Anhui Normal University, Wuhu 241000, China; Department of Physics, Anhui Key Laboratory of Optoelectric Materials Science and Technology, Key Laboratory of Functional Molecular Solids, Ministry of Education, Anhui Normal University, Wuhu 241000, China; Department of Physics and Astronomy, Rutgers, The State University of New Jersey, Piscataway, NJ 08854-8019, USA; Department of Physics, Anhui Key Laboratory of Optoelectric Materials Science and Technology, Key Laboratory of Functional Molecular Solids, Ministry of Education, Anhui Normal University, Wuhu 241000, China; National Laboratory for Physical Sciences at Microscale and Department of Modern Physics, University of Science and Technology of China, Hefei 230026, China; Department of Physics and Electronic Information Engineering, Minjiang University, Fuzhou 350108, China

**Keywords:** critical phenomena, universality class, O(*n*) vector model, finite-size scaling

## Abstract

Logarithmic finite-size scaling of the O(*n*) universality class at the upper critical dimensionality (*d*_*c*_ = 4) has a fundamental role in statistical and condensed-matter physics and important applications in various experimental systems. Here, we address this long-standing problem in the context of the *n*-vector model (*n* = 1, 2, 3) on periodic four-dimensional hypercubic lattices. We establish an explicit scaling form for the free-energy density, which simultaneously consists of a scaling term for the Gaussian fixed point and another term with multiplicative logarithmic corrections. In particular, we conjecture that the critical two-point correlation *g*(*r*, *L*), with *L* the linear size, exhibits a two-length behavior: follows $r^{2-d_c}$ governed by the Gaussian fixed point at shorter distances and enters a plateau at larger distances whose height decays as $L^{-d_c/2}({\rm ln}L)^{\hat{p}}$ with $\hat{p}=1/2$ a logarithmic correction exponent. Using extensive Monte Carlo simulations, we provide complementary evidence for the predictions through the finite-size scaling of observables, including the two-point correlation, the magnetic fluctuations at zero and nonzero Fourier modes and the Binder cumulant. Our work sheds light on the formulation of logarithmic finite-size scaling and has practical applications in experimental systems.

## INTRODUCTION

The O(*n*) model of interacting vector spins is a much-applied model in condensed-matter physics and one of the most significant classes of lattice models in equilibrium statistical mechanics [1,2]. The Hamiltonian of the O(*n*) vector model is written as

(1) \begin{equation*} \mathcal {H}=-\sum \limits _{\langle {\bf r}{\bf r^{\prime }}\rangle }\vec{S}_{\bf r} \cdot \vec{S}_{\bf r^{\prime }}, \end{equation*}

where $\vec{S}_{\bf r}$ is an *n*-component isotropic spin with unit length and the summation runs over nearest neighbors. Prominent examples include the Ising (*n* = 1), XY (*n* = 2) and Heisenberg (*n* = 3) models of ferromagnetism, as well as the self-avoiding random walk (*n* → 0) in polymer physics. Its experimental realization is now available for various *n* values in magnetic materials [3–7], superconducting arrays [8,9] and ultracold atomic systems [10,11].

Finite-size scaling (FSS) is an extensively utilized method for studying systems of continuous phase transitions [12], including the O(*n*) vector model (1). Near criticality, these systems are characterized by a diverging correlation length ξ ∝ *t*^−ν^, where the parameter *t* measures the deviation from the critical point and ν is a critical exponent. For a finite box with linear size *L*, the standard FSS hypothesis assumes that ξ is bounded by the linear size *L*, and thus predicts that the singular part *f*(*t*, *h*) of the free-energy density scales as

(2) \begin{equation*} f(t,h) = L^{-d} \tilde{f} (t L^{y_t}, hL^{y_h}), \end{equation*}

where $\tilde{f}$ is a universal scaling function, *t* and *h* represent the thermal and magnetic scaling fields, and *y*_*t*_ = 1/ν and *y*_*h*_ are the corresponding thermal and magnetic renormalization exponents, respectively. Furthermore, the standard FSS theory hypothesizes that, at criticality, the spin-spin correlation function $g(r,L) \equiv \langle \vec{S}_0 \cdot \vec{S}_{\bf r} \rangle$ of distance *r* decays as

(3) \begin{equation*} g(r,L) \asymp r^{-(d-2+\eta )}\tilde{g} (r/L), \end{equation*}

where η relates to *y*_*h*_ by the scaling relation η = 2 + *d* − 2*y*_*h*_. From (2) and (3), the FSS of various macroscopic physical quantities can be obtained. For instance, from the second derivative of *f*(*t*, *h*) with respect to *t* or *h*, it follows that, at criticality, the specific heat behaves as $C \asymp L^{2y_t-d}$ and the magnetic susceptibility diverges as $\chi \asymp L^{2y_h-d}$. The FSS of χ can also be calculated by summing *g*(*r*, *L*) over the system. Furthermore, the thermodynamic critical exponents can be obtained by the (hyper-)scaling relations. For instance, in the thermodynamic limit (*L* → ∞), the specific heat and the magnetic susceptibility scale as *C* ∝ *t*^−α^ and χ ∝ *t*^−γ^, where the critical exponents are α = 2 − *d*/*y*_*t*_ and γ = (2*y*_*h*_ − *d*)/*y*_*t*_.

The O(*n*) model exhibits an upper critical dimensionality *d*_*c*_ = 4 such that the thermodynamic scaling in higher dimensions *d* > *d*_*c*_ are governed by the Gaussian fixed point, which has the critical exponents α = 0 and γ = 1, etc. In the framework of the renormalization group, the renormalization exponents near the Gaussian fixed point are

(4) \begin{equation*} y_t = 2 \quad \mbox{and} \quad y_h = 1 + d/2 \end{equation*}

for *d* > *d*_*c*_.

Accordingly, the standard FSS formulae (2) and (3) predict that the critical susceptibility diverges as $\chi \asymp L^{2y_h-d} = L^2$ for *d* > *d*_*c*_. However, for the Ising model on 5D periodic hypercubes, χ was numerically observed to scale as *L* ≍ *L*^5/2^ instead of *L*^2^ [13–18]. The FSS for *d* ≥ *d*_*c*_ turns out to be surprisingly subtle and remains a topic of extensive controversy [13–21].

It was realized that, for *d* > *d*_*c*_, the Gaussian exponents *y*_*t*_ and *y*_*h*_ in (4) can be renormalized by the leading irrelevant thermal field with exponent *y*_*u*_ = 4 − *d* as [22–25]

(5) \begin{equation*} y_t^{*}=y_t-\frac{y_u}{2} = \frac{d}{2} \quad\!\! \mbox{and} \quad\!\! y_h^{*}=y_h-\frac{y_u}{4} =\frac{3d}{4}, \end{equation*}

and the FSS of the free-energy density *f*(*t*, *h*) becomes

(6) \begin{equation*} f(t,h) = L^{-d} \tilde{f} (t L^{y_t^{*}}, hL^{y_h^{*}}). \end{equation*}

In this scenario of the dangerously irrelevant field, the FSS of the critical susceptibility becomes $\chi \asymp L^{2y_h^*-d} = L^{d/2}$, consistent with the numerical results [13–15,17,18]. It was further assumed that the scaling behavior of *g*(*r*, *L*) is modified as [16]

(7) \begin{equation*} g(r,L) \asymp r^{-(d-2+\eta _Q)} \tilde{g}(r/L) \end{equation*}

with η_*Q*_ = 2 − *d*/2, such that the decay of *g*(*r*, *L*) is no longer Gaussian-like. In the study of the 5D Ising model [13], a more subtle scenario was proposed that *g*(*r*, *L*) decays as *r*^−3^ at short distances, gradually becomes *r*^−5/2^ for large distances and has a crossover behavior in between. The introduction of η_*Q*_ was refuted by Wittmann and Young [15] as the magnetic fluctuations at nonzero Fourier mode **k** ≠ 0 scale as χ_**k**_ ≍ *L*^2^ and underlined in [17], which revealed that the nonzero Fourier moments are governed by the Gaussian fixed point instead of being contaminated by the dangerously irrelevant field.

Using random-current and random-path representations [26–28], Papathanakos [19] conjectured that the scaling behavior of *g*(*r*, *L*) has a two-length form as

(8) \begin{equation*} g(r,L) \asymp \left\lbrace \begin{array}{@{}l@{\quad }l@{}}r^{-(d-2)}, & r \le \mathcal {O}(L^{d/[2(d-2)]}) ,\\ L^{-d/2}, & r \ge \mathcal {O}(L^{d/[2(d-2)]}). \end{array}\right. \end{equation*}

According to (8), the critical correlation function still exhibits a Gaussian-like decay, *g*(*r*, *L*) ≍ *r*^−(*d* − 2)^, up to a length scale ξ_1_ = *L*^*d*/[2(*d* − 2)]^, and then enters an *r*-independent plateau whose height vanishes as *L*^−*d*/2^. Since the length ξ_1_ is vanishingly small compared to the linear size, ξ_1_/*L* → 0, the plateau effectively dominates the scaling behavior of *g*(*r*, *L*) and the FSS of χ. The two-length scaling form (8) has been numerically confirmed for the 5D Ising model and self-avoiding random walk, with a geometric explanation based on the introduction of an unwrapped length on the torus [18]. It is also consistent with the rigorous calculations for the so-called random-length random-walk model [20]. It is noteworthy that the two-length scaling is able to explain both the FSS χ_0_ ≡ χ ≍ *L*^5/2^ for the susceptibility (the magnetic fluctuations at the zero Fourier mode) [14] and the FSS χ_**k**_ ≍ *L*^2^ for the magnetic fluctuations at nonzero modes [15,17].

Combining all the existing numerical and (semi-)analytical insights  [13–20], Y.D. and coworkers extended the scaling form (6) for the free energy to be [21]

(9) \begin{eqnarray*} f(t,h) &=& L^{-d} \tilde{f}_0 (t L^{y_t}, hL^{y_h})\nonumber\\&&+\,L^{-d} \tilde{f}_1 (t L^{y^*_t}, hL^{y^*_h}), \end{eqnarray*}

where (*y*_*t*_, *y*_*h*_) are the Gaussian exponents (4) and $(y_t^{*},y_h^{*})$ are still given by (5). Conceptually, scaling formula (9) explicitly points out the coexistence of two sets of exponents (*y*_*t*_, *y*_*h*_) and $(y_t^{*},y_h^{*})$, which was implied in previous studies [15,17,18,20]. Moreover, a simple perspective of understanding was provided [21] that the scaling term with $\tilde{f}_1$ can be regarded as corresponding to the FSS of the critical O(*n*) model on a finite complete graph with *V* = *L*^*d*^ vertices. As a consequence, the exponents $(y_t^{*},y_h^{*})$ can be directly obtained from exact calculations of the complete-graph O(*n*) vector model, which also gives $y_t^{*}=d/2$ and $y_h^{*}=3d/4$. From this correspondence, the plateau of *g*(*r*, *L*) in (8) is in line with the FSS of the complete-graph correlation function $g_{i\ne j} \equiv $$\langle \vec{S}_i \cdot \vec{S}_j \rangle$, which also decays as *V*^−1/2^ = *L*^−*d*/2^. Note that, as a counterpart of the complete-graph scaling function, the term with $\tilde{f}_1$ should not describe the FSS of quantities merely associated with *r*-dependent behaviors, including magnetic/energylike fluctuations at nonzero Fourier modes. Therefore, in comparison with (6), scaling formula (9) can give the FSS of a more exhaustive list of physical quantities. The following gives some examples at criticality.

- Let $\vec{\cal M} \equiv \sum _{\bf r} \vec{S}_{\bf r}$ specify the total magnetization of a spin configuration, and measure its ℓ moment as $M_{\ell } \equiv \langle |\vec{\cal M}|^\ell \rangle$. Equation (9) predicts that $M_{\ell } \sim L^{\ell y^*_h}+q L^{\ell y_h}$, with *q* a nonuniversal constant. In particular, the magnetic susceptibility $\chi _0 \equiv L^{-d} M_2 \asymp L^{d/2} [1+\mathcal {O}(L^{(4-d)/2})]$, where the FSS from the Gaussian term $\tilde{f}_0$ is effectively a finite-size correction, but its existence is important in analyzing numerical data [21].
- Let $\vec{\cal M}_{\bf k} \equiv \sum _{\bf r} \vec{S}_{\bf r} e^{i {\bf k} \cdot {\bf r}}$ specify the Fourier mode of magnetization with momentum **k** ≠ 0, and measure its ℓ moment as $M_{\ell , {\bf k}} \equiv \langle |\vec{\cal M}_{\bf k} |^\ell \rangle$. The magnetic fluctuations at **k** ≠ 0 behave as $\chi _{\bf k} \equiv L^{-d} M_{2, {\bf k} } \sim L^{2y_h-d}=L^2$. The behaviors of χ_0_ and χ_**k**_ have been confirmed for the 5D Ising model [15,17,18,20].
- The Binder cumulant $Q \equiv \langle |\vec{\cal M} |^2 \rangle ^2/\break\langle |\vec{\cal M} |^4 \rangle$ should take the complete-graph value, as expected from the correspondence between the term with $\tilde{f}_1$ in (9) and the complete-graph FSS. For the Ising model, the complete-graph calculations give *Q* = 4[Γ(3/4)/Γ(1/4)]^2^ ≈ 0.456 947, consistent with the 5D result in [13].

Analogously, the FSS behaviors of the energy density, its higher-order fluctuations and the ℓ-moment Fourier modes at **k** ≠ 0 can be derived from (9).

We expect that the FSS formulae (8) and (9) are valid not only for the O(*n*) vector model but also for generic systems of continuous phase transitions at *d* > *d*_*c*_. An example is given for percolation that has *d*_*c*_ = 6. It was observed [29] that, at criticality, the probability distributions of the largest-cluster size follow the same scaling function for 7D periodic hypercubes and on the complete graph.

In this work, we focus on the FSS for the O(*n*) vector model at the upper critical dimensionality *d* = *d*_*c*_. In this marginal case, it is known that multiplicative and additive logarithmic corrections would appear in the FSS. However, exploring these logarithmic corrections turns out to be notoriously hard. The challenge comes from the lack of analytical insights, the existence of slow finite-size corrections, as well as the unavailability of very large system sizes in simulations of high-dimensional systems.

For the O(*n*) vector model, establishing the precise FSS form at *d* = *d*_*c*_ is not only of fundamental importance in statistical mechanics and condensed-matter physics, but also of practical relevance due to the direct experimental realizations of the model, particularly in three-dimensional quantum critical systems [3–6,10,11]. For instance, to explore the stability of Anderson–Higgs excitation modes in systems with continuous symmetry breaking (*n* ≥ 2), a crucial theoretical question is whether or not the Gaussian *r*-dependent behavior *g*(*r*) ≍ *r*^−2^ is modified by some multiplicative logarithmic corrections.

## SUMMARY OF THE MAIN FINDINGS

At the upper critical dimensionality (*d*_*c*_ = 4) of the O(*n*) model, state-of-the-art applications of FSS are mostly restricted to a phenomenological scaling form proposed by Kenna [30] for the singular part of the free-energy density, which was extended from Aktekin’s formula for the Ising model [31],

(10) \begin{equation*} f(t,h)=L^{-4} \tilde{f} (t L^{y_t} ({\rm ln}L)^{\hat{y}_t},hL^{y_h} ({\rm ln}L)^{\hat{y}_h}) \end{equation*}

for *n* ≥ 0 and *n* ≠ 4, where the renormalization exponents *y*_*t*_ = 2 and *y*_*h*_ = 3 are given by (4). Furthermore, the renormalization-group calculations predicted the logarithmic-correction exponents as $\hat{y}_t={(4-n)}/{(2n+16)}$ and $\hat{y}_h=1/4$ [32,33]. The leading FSS of χ_0_ is hence given by χ_0_ ≍ *L*^2^(ln*L*)^1/2^, independent of *n*.

Motivated by recent progress in O(*n*) models for *d* > *d*_*c*_ [15–21], we hereby propose that, at *d* = *d*_*c*_, the scaling form (10) for the free energy should be revised as

(11) \begin{eqnarray*} f(t,h)&=& L^{-4} \tilde{f}_0 (t L^{y_t}, hL^{y_h})\nonumber\\ && + L^{-4} \tilde{f}_1(t L^{y_t} (\ln L)^{\hat{y}_t}, hL^{y_h} (\ln L)^{\hat{y}_h} ),\nonumber\\ \end{eqnarray*}

and the critical two-point correlation *g*(*r*, *L*) behaves as

(12) \begin{equation*} g(r,L) \asymp \left\lbrace \begin{array}{@{}l@{\quad }l@{}}r^{-2}, & r \le \mathcal {O}(L/({\rm ln}L)^{\hat{p}} ),\\ L^{-2}({\rm ln}L)^{\hat{p}}, & r \ge \mathcal {O}(L/({\rm ln}L)^{\hat{p}}),\end{array}\right. \end{equation*}

with $\hat{p} = 2 \hat{y}_h = 1/2$. By (12), we explicitly point out that no multiplicative logarithmic correction appears in the *r* dependence of *g*(*r*, *L*) ≍ *r*^−2^, which is still Gaussian-like. By contrast, the plateau for $r \ge \xi _1 \sim L/({\rm ln}L)^{\hat{p}}$ is modified as $L^{-2}({\rm ln}L)^{\hat{p}}$. In other words, along any direction of the periodic hypercube, we have $g(r,L) \asymp r^{-2} + v L^{-2}({\rm ln}L)^{\hat{p}}$, with $v$ a nonuniversal constant. The *r*^−2^ decay at shorter distances in (12) is consistent with analytical calculations for the 4D weakly self-avoiding random walk and the O(*n*) φ^4^ model directly in the thermodynamic limit (*L* → ∞) [34], which predict $g(r) \asymp r^{-2} (1 + \mathcal {O}(1/{\rm ln} r))$.

The roles of terms with $\tilde{f}_0$ and $\tilde{f}_1$ in (11) are analogous to those in (9). The former arises from the Gaussian fixed point, and the latter describes the ‘background’ contributions (**k** = 0) for the FSS of macroscopic quantities. However, note that the term with $\tilde{f}_1$ can no longer be regarded as an exact counterpart of the FSS of the complete graph, due to the existence of multiplicative logarithmic corrections. By contrast, the exact complete-graph mechanism applies to the $\tilde{f}_1$ term in (9), where the logarithmic correction is absent and $\tilde{f}_1$ corresponds to the free energy of the standard complete-graph model. According to (11), the FSS of various macroscopic quantities at *d* = *d*_*c*_ can be obtained as follows.

- The magnetization density $m \equiv L^{-d} \langle |\vec{\cal M}|\rangle \asymp L^{-1} (\ln L)^{\hat{y}_h} [1+\mathcal {O}((\ln L)^{-\hat{y}_h}) ]$.
- The magnetic susceptibility $\chi _0 \asymp L^{2}(\ln L)^{2 \hat{y}_h} [1+\mathcal {O}((\ln L)^{-2\hat{y}_h}) ]$.
- The magnetic fluctuations at **k** ≠ 0 Fourier modes χ_**k**_ ≍ *L*^2^.
- The Binder cumulant *Q* may not take the exact complete-graph value, due to the multiplicative logarithmic correction. Some evidence was observed in a recent study by Y.D. and his coworkers for the self-avoiding random walk (*n* = 0) on 4D periodic hypercubes, in which the maximum system size is up to *L* = 700.

The FSS of the energy density, its higher-order fluctuations and the ℓ-moment Fourier modes at **k** ≠ 0 can be obtained.

In quantities like *m* and χ_0_, the FSS from the Gaussian fixed point effectively plays the role of finite-size corrections. Nevertheless, we note that in the analysis of numerical data, it is important to include such scaling terms.

We remark that the FSS formulae (11) and (12) for *d* = *d*_*c*_ are less generic than (8) and (9) for *d* > *d*_*c*_. For the O(*n*) models, a multiplicative logarithmic correction is absent in the Gaussian *r* dependence of *g*(*r*, *L*) in (12). Although the two length scales are possibly generic features of models with logarithmic finite-size corrections at upper critical dimensionality, multiplicative logarithmic corrections to the *r* dependence of *g*(*r*, *L*) require case-by-case analyses. Equation (11) can be modified in some of these models, which include the percolation and spin-glass models in six dimensions.

**Figure 1.**
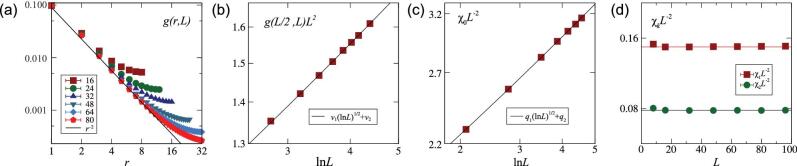
Evidence for conjectured formulae (11) and (12) in the example of the critical four-dimensional (4D) XY model. (a) Correlation function *g*(*r*, *L*) on a log-log scale. The solid line denotes *r*^−2^ behavior. (b) Scaled correlation *g*(*r*, *L*)*L*^2^ with *r* = *L*/2 versus ln*L* on a log-log scale. Thus, the horizontal axis is effectively on a double logarithmic scale of *L*. The solid line represents logarithmic divergence with $\hat{p} = 1/2$. (c) Scaled magnetic susceptibility χ_0_*L*^−2^ versus ln*L* on a log-log scale. The solid line accounts for logarithmic divergence with $\hat{p} = 1/2$. (d) Scaled **k** ≠ 0 magnetic fluctuations χ_1_*L*^−2^ and χ_2_*L*^−2^, with **k**_1_ = (2π/*L*, 0, 0, 0) and **k**_2_ = (2π/*L*, 2π/*L*, 0, 0), respectively. The horizontal lines strongly indicate the absence of logarithmic corrections in the scaling of χ_**k**_.

We proceed to verify (11) and (12) using extensive Monte Carlo (MC) simulations of the O(*n*) vector model. Before giving the technical details, in Fig. 1 we present complementary evidence for (11) and (12) in the case of the critical 4D XY model. In Fig. 1(a) we show the extensive data of *g*(*r*, *L*) for 16 ≤ *L* ≤ 80, of which the largest system contains about 4 × 10^7^ lattice sites. To demonstrate the multiplicative logarithmic correction in the large-distance plateau indicated by (12), we plot *g*(*L*/2, *L*)*L*^2^ versus ln*L* on a log-log scale in Fig. 1(b). The excellent agreement between the MC data and the formula $v$_1_(ln*L*)^1/2^ + $v$_2_ provides a first piece of evidence for the presence of the logarithmic correction with exponent $\hat{p} = 1/2$. The second piece of evidence comes from Fig. 1(c), which suggests that the χ_0_*L*^−2^ data can be well described by the formula *q*_1_(ln*L*)^1/2^ + *q*_2_. Finally, in Fig. 1(d) we plot the **k** ≠ 0 magnetic fluctuations χ_1_ and χ_2_ with **k**_1_ = (2π/*L*, 0, 0, 0) and **k**_2_ = (2π/*L*, 2π/*L*, 0, 0), respectively, which suppress the *L*-dependent plateau and show the *r*-dependent behavior of *g*(*r*, *L*). Indeed, the χ_1_*L*^−2^ and χ_2_*L*^−2^ data converge rapidly to constants as *L* increases.

## NUMERICAL RESULTS AND FINITE-SIZE SCALING ANALYSES

Using a cluster MC algorithm [35], we simulate Hamiltonian (1) on 4D hypercubic lattices up to *L*_max_ = 96 (Ising, XY) and 56 (Heisenberg), and measure a variety of macroscopic quantities, including the magnetization density *m*, the susceptibility χ_0_, the magnetic fluctuations χ_1_ and χ_2_ and the Binder cumulant *Q*. Moreover, we compute the two-point correlation function *g*(*r*, *L*) for the XY model up to *L*_max_ = 80 by means of a state-of-the-art worm MC algorithm [36].

### Estimates of critical temperatures

In order to locate the critical temperatures *T*_*c*_, we perform least-squares fits for the finite-size MC data of the Binder cumulant to

(13) \begin{eqnarray*} Q(L,T)&=&Q_c+atL^{y_t}({\rm ln}L)^{\hat{y}_t}+b({\rm ln} L)^{-\hat{p}}\nonumber\\ &&+\,c \frac{{\rm ln}({\rm ln}L)}{{\rm ln}L}, \end{eqnarray*}

where *t* is explicitly defined as *T*_*c*_ − *T*, *Q*_*c*_ is a universal ratio, and *a*, *b*, *c* are nonuniversal parameters. In addition to the leading additive logarithmic correction, we include *c*(ln(ln*L*))/ln*L* proposed by Kenna [30] as a high-order correction, ensuring the stability of fits. In all fits, we justify the confidence in a standard manner: the fits with Chi squared (χ^2^) per degree of freedom (DF) is $\mathcal {O}(1)$ and remains stable as the cutoff size *L*_min_ increases. The latter is a caution against possible high-order corrections not included. The details of the fits are presented in the online supplementary material.

**Figure 2.**
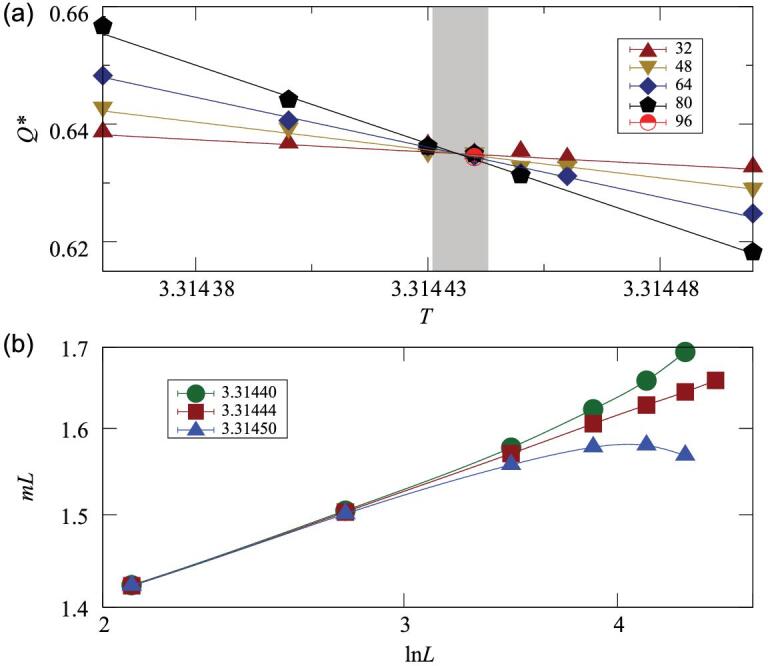
Locating *T*_*c*_ for the 4D XY model. (a) The Binder cumulant *Q* with finite-size corrections being subtracted, namely, Q^*^(*L*, *T*) = *Q*(*L*, *T*) − *b*(ln*L*)^−1/2^, with *b* ≈ 0.1069 according to a preferred least-squares fit. The shadow marks *T*_*c*_ and its error margin. (b) The magnetization density *m* rescaled by *L*^−1^ versus ln*L* around *T*_*c*_ = 3.314 44 on a log-log scale.

By analyzing the finite-size correction *Q*(*L*, *T*_*c*_) − *Q*_*c*_, we find that the leading correction is nearly proportional to (ln*L*)^−1/2^, consistent with the prediction of (11) and (12). We let *Q*_*c*_ be free in the fits and have *Q*_*c*_ = 0.45(1), close to the complete-graph result *Q*_*c*_ = 0.456 947. Besides, we perform simulations for the XY and Heisenberg models on the complete graph and obtain *Q*_*c*_ ≈ 0.635 and 0.728, respectively, also close to the fitting results of the 4D *Q* data. We obtain *T*_*c*_(XY) = 3.314 437(6), and in Fig. 2(a) we illustrate the location of *T*_*c*_ by *Q*.

We further examine the estimate of *T*_*c*_ by the FSS of other quantities, such as the magnetization density *m*. For the XY model, in Fig. 2(b) we give a log-log plot of the *mL* data versus ln*L* for *T* = *T*_*c*_, as well as for *T*_low_ = 3.314 40 and *T*_above_ = 3.314 50. The significant bending-up and bending-down features clearly suggest that *T*_low_ < *T*_*c*_ and *T*_above_ > *T*_*c*_, providing confidence for the finally quoted error margin of *T*_*c*_.

The final estimates of *T*_*c*_ are summarized in Table 1. For *n* = 1, we have *T*_*c*_ = 6.680 300(10), which is consonant with and improves over *T*_*c*_ = 6.680 263(23) [37] and marginally agrees with *T*_*c*_ = 6.679 63(36) [38] and 6.680 339(14) [13]. For *n* = 2, our determination *T*_*c*_ = 3.314 437(6) significantly improves over *T*_*c*_ = 3.31 [39,40] and 3.314 [41]. For *n* = 3, our result *T*_*c*_ = 2.198 79(2) rules out *T*_*c*_ = 2.192(1) from a high-temperature expansion [42].

**Table 1.** Estimates of *T*_*c*_ for the 4D O(*n*) vector models.

Model	*T*_*c*_	Reference
Ising (*n* = 1)	6.679 63(36)	[38]
	6.680 339(14)	[13]
	6.680 263(23)	[37]
	6.680 300(10)	This work
XY (*n* = 2)	3.31 and 3.314	[39–41]
	3.314 437(6)	This work
Heisenberg (*n* = 3)	2.192(1)	[42]
	2.198 79(2)	This work

### Finite-size scaling of the two-point correlation

We then fit the critical two-point correlation *g*(*L*/2, *L*) to

(14) \begin{equation*} g(L/2,L) = v_1 L^{-2}({\rm ln}L)^{\hat{p}} + v_2 L^{-2}, \end{equation*}

where the first term comes from the large-distance plateau and the second term comes from the *r*-dependent behavior of *g*(*r*, *L*). With $\hat{p}=1/2$ being fixed, the estimate of the leading scaling term *L*^−1.98(4)^ agrees well with the exact *L*^−2^. With the exponent −2 in *L*^−2^ being fixed, the result $\hat{p}=0.5(1)$ is also well consistent with the prediction $\hat{p}=1/2$. These results are elaborated in the online supplementary material.

We remark that FSS analyses for *g*(*L*/2, *L*) have already been performed in [16] with the formula *g*(*L*/2, *L*) = *AL*^−2^[ln(*L*/2 + *B*)]^1/2^ (*A* and *B* are constants) and in [13] with a similar formula. These FSSs in the literature correspond to the first scaling term in (14). Hence, (14) serves as a forward step for complete FSS by involving the scaling term $v$_2_*L*^−2^, which arises from the Gaussian fixed point.

### Finite-size scaling of the magnetic susceptibility

According to (11) and (12), we fit the critical susceptibility χ_0_ to

**Figure 3.**
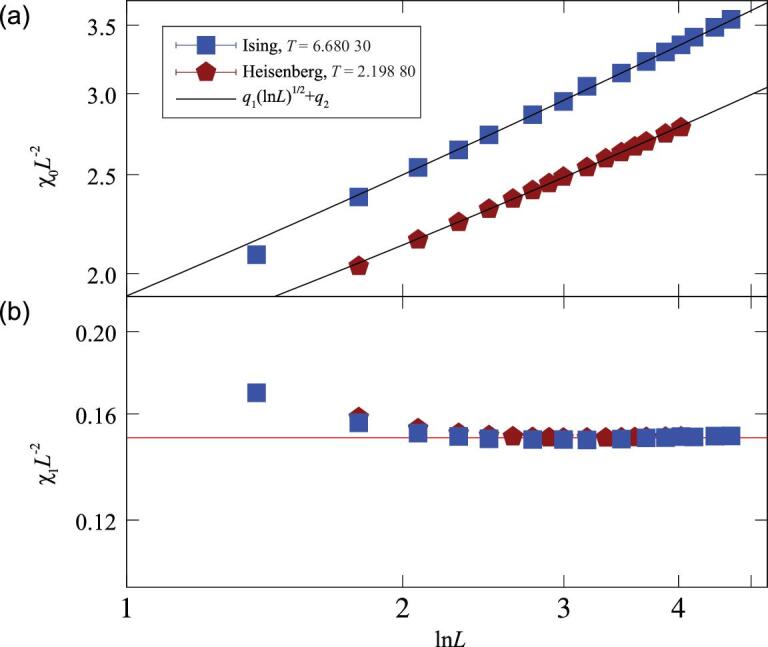
The magnetic fluctuations (a) χ_0_ and (b) χ_1_ rescaled by *L*^2^ versus ln*L* on a log-log scale for the critical Ising and Heisenberg models. The black lines in (a) represent the least-squares fits, and the red line in (b) denotes a constant.

(15) \begin{equation*} \chi _0 = q_1 L^{2}({\rm ln}L)^{\hat{p}}+ q_2 L^{2} \end{equation*}

with *q*_1_ and *q*_2_ nonuniversal constants. For $\hat{p}=1/2$ being fixed, we obtain fitting results with χ^2^/DF ≲ 1 for each *n* = 1, 2, 3, and correctly produce the leading scaling form *L*^2^. The scaled susceptibility χ_0_*L*^−2^ versus ln*L* is shown in Fig. 1(c) for the XY model and in Fig. 3(a) for the Ising and Heisenberg models.

We note that previous studies based on a FSS without high-order corrections produced estimates of ${\hat{y}}_h$$(=\hat{p}/2)$, considered to be consistent with ${\hat{y}}_h=1/4$ [38,43–45]. The maximum lattice size therein was *L*_max_ = 24, four times smaller than *L*_max_ = 96 of the present study. In particular, it was reported [43] that $2\hat{y}_h=0.45(8)$ and $4\hat{y}_h=0.80(25)$. Nevertheless, we find that the fit $\chi _0= q_1 L^2 ({\rm ln}L)^{2 \hat{y}_h}$ by dropping the correction term *q*_2_*L*^2^ would yield $\hat{y}_h=0.21(1)$ (Ising), 0.20(1) (XY), and 0.19(1) (Heisenberg), which are smaller than and inconsistent with the predicted value $\hat{y}_h=1/4$. This suggests the significance of *q*_2_*L*^2^ in the susceptibility χ_0_, which arises from the *r* dependence of *g*(*r*, *L*).

### Finite-size scaling of the magnetic fluctuations at nonzero Fourier modes

We consider the magnetic fluctuations χ_1_ with |**k**_1_| = 2π/*L* and χ_2_ with $|{\bf k}_2|= 2 \sqrt{2} \pi /L$. We have compared the FSSs of χ_0_, χ_1_ and χ_2_ in Fig. 1(c) and (d) for the critical 4D XY model. As *L* increases, χ_1_*L*^−2^ and χ_2_*L*^−2^ converge rapidly, suggesting the absence of a multiplicative logarithmic correction. This is in sharp contrast to the behavior of χ_0_*L*^−2^, which diverges logarithmically. For the Ising and Heisenberg models, the FSS of the fluctuations at nonzero modes is also free of a multiplicative logarithmic correction (Fig. 3(b)).

Surprisingly, we find that the scaled fluctuations χ_1_*L*^−2^ ≈ 0.15 are equal within error bars for the Ising, XY and Heisenberg models.

**Figure 4.**
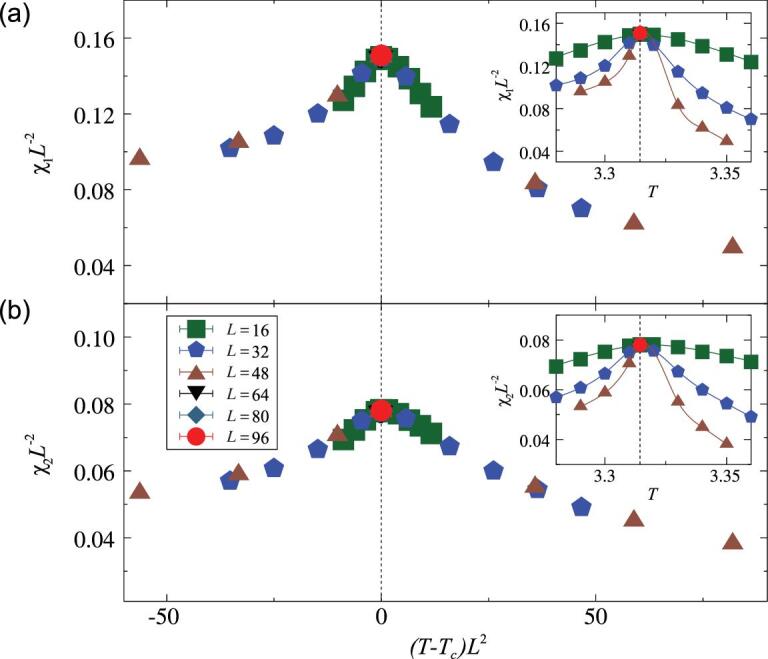
Data collapses for the magnetic fluctuations (a) χ_1_ and (b) χ_2_ rescaled by $L^{2 y_h-d}$ and $L^{y_t}$ (*y*_*h*_ = 3, *y*_*t*_ = 2, *d* = 4) for the 4D XY model. The insets show the scaled fluctuations versus *T*, and the dashed lines denote *T*_*c*_.

Furthermore, we show in Fig. 4 χ_1_ and χ_2_ versus *T* for the 4D XY model. We observe that the magnetic fluctuations at nonzero Fourier modes reach maximum at *T*_*c*_ and that the χ_1_*L*^−2^ (χ_2_*L*^−2^) data for different *L*s collapse well not only at *T*_*c*_ but also for a wide range of $(T-T_c)L^{y_t}$ with *y*_*t*_ = 2.

## DISCUSSIONS

We propose formulae (11) and (12) for the FSS of the O(*n*) universality class at the upper critical dimensionality, which are tested against extensive MC simulations with *n* = 1, 2, 3. From the FSS of the magnetic fluctuations at zero and nonzero Fourier modes, the two-point correlation function and the Binder cumulant, we obtain complementary and solid evidence supporting (11) and (12). As byproducts, the critical temperatures for *n* = 1, 2, 3 are all located up to an unprecedented precision.

An immediate application of (12) is to the massive amplitude excitation mode (often called the Anderson–Higgs boson) due to the spontaneous breaking of the continuous O(*n*) symmetry [46], which is at the frontier of condensed matter research. At the pressure-induced quantum critical point (QCP) in the dimerized quantum antiferromagnet TlCuCl_3_, the 3D O(3) amplitude mode was probed by neutron spectroscopy and a rather narrow peak width of about 15% of the excitation energy was revealed, giving no evidence for the logarithmic reduction of the width-mass ratio [3]. This was later confirmed by a quantum MC study of a 3D model Hamiltonian of O(3) symmetry [5,6]. Indeed, (12) provides an explanation why the logarithmic-correction reduction in the Higgs resonance was not observed at the 3D QCP. In numerical studies of the Higgs excitation mode at the 3D QCP, the correlation function *g*(τ ≡ |τ_1_ − τ_2_|) is measured along the imaginary-time axis β, and numerical analytical continuation is used to deal with the *g*(τ) data. In practice, simulations are carried out at very low temperature β → ∞, and it is expected that *g*(τ) ≍ τ^−2^ for a significantly wide range of τ. Furthermore, it is the τ-dependent behavior of *g*(τ), instead of the *L* dependence, that plays a decisive role in numerical analytical continuation.

In the thermodynamic limit, the two-point correlation function decays as $g(r) \sim r^{-2} \tilde{g}(r/\xi )$, where the scaling function $\tilde{g}(r/\xi )$ quickly drops to zero as *r*/ξ ≫ 1. It can be seen that no multiplicative logarithmic correction exists in the algebraic decaying behavior. On the other hand, as the criticality is approached (*t* → 0), the correlation length diverges as $\xi (t) \sim t^{-1/2}|{\rm ln}t|^{\hat{\nu }}$, and $\hat{\nu } = {(n+2)}/{2(n+8)} > 0$ implies that ξ diverges faster than *t*^−1/2^ [30,33]. Since the susceptibility can be calculated by summing the correlation as $\chi _0 \sim \int _0^{\xi } g(r)r^{d-1}dr \sim \xi ^2$, we have $\chi _0(t) \sim t^{-1} |\ln t|^{\hat{\gamma }}$ with $\hat{\gamma } = 2 \hat{\nu }$. The thermodynamic scaling of χ_0_(*t*) can also be obtained from the FSS formula (10) or (11), which gives $\chi _0(t,L) \sim L^{2y_h-4} (\ln L)^{2\hat{y}_h} \tilde{\chi _0} (tL^{y_t} (\ln L)^{\hat{y}_t})$. By fixing $tL^{y_t} (\ln L)^{\hat{y}_t}$ at some constant, we obtain the relation $L \sim t^{-1/y_t} |\ln t|^{-\hat{y}_t/y_t}$. Substituting this into the FSS of χ_0_(*t*, *L*) yields $\chi _0(t) \sim t^{\gamma } |\ln t|^{\hat{\gamma }}$ with γ = (2*y*_*h*_ − 4)/*y*_*t*_ and $\hat{\gamma } = -\gamma \hat{y}_t+2\hat{y}_h$. With $(y_t, y_h, \hat{y}_t, \hat{y}_h)\,=\,(2, 3,\, {(4-n)}/{(2n+16)}, \frac{1}{4})$, we have γ = 1 and $\hat{\gamma } = {(n+2)}/{(n+8)}$. The thermodynamic scaling with logarithmic corrections has been demonstrated in [4] in terms of the magnetization *m* of an O(3) Hamiltonian.

For the critical Ising model in five dimensions, an unwrapped distance *r*_u_ was introduced to account for the winding numbers across a finite torus [18]. The unwrapped correlation was shown to behave as $g(r_{\rm u}) \sim r_{\rm u}^{2-d} \tilde{g} (r_{\rm u}/\xi _{\rm u})$, where the unwrapped correlation length diverges as ξ_u_ ∼ *L*^*d*/4^. This differs from typical correlation functions that are cut off by a linear system size of approximately *L*. We expect that at *d*_*c*_ = 4 the unwrapped correlation length diverges as $\xi _{\rm u} \sim L (\ln L)^{\hat{y}_h}$, which gives the critical susceptibility as $\chi _0(L) \sim L^2 (\ln L)^{2\hat{y}_h}$.

Besides, (12) is useful for predicting various critical behaviors. As an instance, it was observed that an impurity immersed in a 2D O(2) quantum critical environment can evolve into a quasiparticle of fractionalized charge, as the impurity-environment interaction is tuned to a boundary critical point [47–49]. Equation (12) precludes the emergence of such a quantum-fluctuation-induced quasiparticle at the 3D O(2) QCP.

We mention an open question about the specific heat of the 4D Ising model. The FSS formula (10) predicts that the critical specific heat diverges as *C* ≍ (ln *L*)^1/3^. By contrast, an MC study demonstrated that the critical specific heat is bounded [37]. The complete scaling form (11) is potentially useful for reconciling the inconsistency.

Finally, it would be possible to extend the present scheme to other systems of critical phenomena, as the existence of upper critical dimensionality is a common feature therein. These systems include the percolation and spin-glass models at their upper critical dimensionality *d*_*c*_ = 6. We leave this for a future study.

## METHODS

Throughout the paper, the raw data for any temperature *T* and linear size *L* are obtained by means of MC simulations, for which the Wolff cluster algorithm [35] and the Prokof’ev–Svistunov worm algorithm [36] are employed complementarily. Both algorithms are state-of-the-art tools in their own territories.

The O(*n*) vector model (1) in its original spin representation is efficiently sampled by the Wolff cluster algorithm, which is the single-cluster version of the widely utilized nonlocal cluster algorithms. The present study uses the standard procedure of the algorithm, as in the original paper [35] where the algorithm was invented. In some situations, we also use the conventional Metropolis algorithm [50] for benchmarks. The macroscopic physical quantities of interest have been introduced in aforementioned sections for the spin representation.

The two-point correlation function for the XY model (*n* = 2) is sampled by means of the Prokof’ev–Svistunov worm algorithm, which was invented for a variety of classical statistical models [36]. By means of a high-temperature expansion, we perform an exact transformation for the original XY spin model to a graphic model in directed-flow representation. We then introduce two defects for enlarging the state space of directed flows. The Markov chain process of evolution is built upon biased random walks of defects, which satisfy the detailed balance condition. It is defined that the evolution hits the original directed-flow state space when the two defects meet at a site. The details for the exact transformation and a step-by-step procedure for the algorithm have been presented in [51].

## Supplementary Material

nwaa212_Supplemental_File
