# Supplementary material for: Finite-size scaling of O(n) systems at the upper critical dimensionality
Source: Natl Sci Rev. 2020 Aug 31;8(3):nwaa212. doi: 10.1093/nsr/nwaa212 (PMC8288422; doi:10.1093/nsr/nwaa212)
Supplement: nwaa212_Supplemental_File [file nwaa212_supplemental_file.pdf]

Supplemental Material for "Finite-size Scaling of O(n) Systems at the Upper Critical Dimensionality"

Jian-Ping Lv,[1,*] Wanwan Xu,[1] Yanan Sun,[1] Kun Chen,[2,†] and Youjin Deng[3,4,‡]

[1] *Department of Physics, Anhui Key Laboratory of Optoelectric Materials Science and Technology, Key Laboratory of Functional Molecular Solids, Ministry of Education, Anhui Normal University, Wuhu, Anhui 241000, China*

[2] *Department of Physics and Astronomy, Rutgers, The State University of New Jersey, Piscataway, New Jersey 08854-8019*

[3] *National Laboratory for Physical Sciences at Microscale and Department of Modern Physics, University of Science and Technology of China, Hefei, Anhui 230026, China*

[4] *Department of Physics and Electronic Information Engineering, Minjiang University, Fuzhou, Fujian 350108, China*

(Dated: June 14, 2020)

We elaborate the quantifications of the finite-size scaling (FSS) mentioned in the main text. Subsequently, we address the location of the critical temperatures T_c, the FSS of the susceptibility χ_0, and the FSS of the two-point correlation $g(r, L)$ with $r = L/2$.

THE ESTIMATE OF T_c

For each of the four-dimensional Ising, XY, and Heisenberg models, the estimate of T_c is achieved by fitting the finite-size Monte Carlo data of the Binder cumulant Q to the scaling ansatz

$$Q(L,T) = Q_c + atL^{y_t}(\ln L)^{\hat{y}_t} + b(\ln L)^{-\hat{p}} + c\frac{\ln(\ln L)}{\ln L} \quad (1)$$

where $t \equiv T_c - T$, for $t \to 0$. Q_c is the critical dimensionless ratio and a, b, c are constants. In the fits, the mean-field thermal exponent is fixed as $y_t = 2$ for reducing uncertainty.

For the Ising model, as shown in Table I, we perform fits with $\hat{y}_t$ being fixed to be $\hat{y}_t = \frac{4-n}{2n+16} = 1/6$ as predicated by renormalization-group calculations [1] or being free. After obtaining an estimate of T_c, we also perform fits right at T_c. Stable fits with $\chi^2/\mathrm{DF} \lessapprox 1$ are achieved for all of these scenarios. As we let Q_c free, it is found $Q_c = 0.45(1)$, which is close to $Q_c = 0.456\,947$ of the complete-graph model [2]. By these fits, we estimate $\hat{p} \approx 0.5$. The fits for Q of the XY and Heisenberg models are summarized in Tables II and III, where the estimates for Q_c are again close to the results $Q_c \approx 0.635$ and 0.728 by Monte Carlo simulations of XY and Heisenberg models on complete graph [3], respectively. Meanwhile, we note that the amplitude of correction $b \approx 0.1$ is sizeable for each of the four-dimensional models. Hence, the prediction of this study on the existence of the finite-size correction $b(\ln L)^{-\hat{p}}$ is further confirmed. This correction form is visualized by Fig. 1 for the Ising, XY, and Heisenberg models. The locating of T_c is shown in Fig. 2.

The final estimates of T_c are determined by comparing the fits, and are $T_c = 6.680\,30(1)$, $3.314\,437(6)$, and $2.198\,79(2)$, for the Ising, XY, and Heisenberg models, respectively. These estimates can be examined independently using the quantities other than Q, e.g., the magnetization density m. Figure 3 displays m rescaled by the mean-field factor L^{y_h-d} ($y_h = 3$, $d = 4$) versus $\ln L$ in a log-log scale around T_c for the Ising, XY, and Heisenberg models. The linearities at T_c in the plots confirm that the estimated T_c are reasonable for the present lattice size scale, while the bending-up or -down features with respect to the linearities suggest the deviations from T_c. For the Ising model, we confirm the linearity at $T_c = 6.680\,30$, and preclude the temperatures $T = 6.680\,263$ and $6.680\,60$ as T_c. For the XY model, we confirm $T_c = 3.314\,44$, and preclude $T_c = 3.314\,40$ and $3.314\,50$. For the Heisenberg model, $T_c = 2.198\,80$ is confirmed and $T_c = 2.198\,67$ and $2.198\,87$ are both precluded.

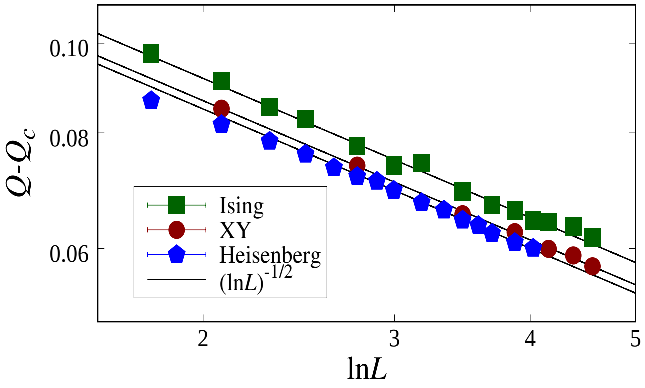

Figure 1. Finite-size corrections $Q(L, T_c) - Q_c$ of the Binder cumulant Q versus $\ln L$ in a log-log scale for the critical Ising, XY, and Heisenberg models. The solid lines are drawn according to preferred fits and stand for the $(\ln L)^{-\frac{1}{2}}$ decaying.

FSS OF THE SUSCEPTIBILITY χ_0

We fit the Monte Carlo data of the susceptibility χ_0 at T_c to the ansatz

$$\chi_0(L, T_c) = q_1 L^{2y_h-d}(\ln L)^{\hat{p}} + q_2 L^{2y_h-d}, \quad (2)$$

which is an inference drawn from the scaling formulae of free energy density and two-point correlation. The fits are summarized in Table IV for the critical Ising, XY, and Heisenberg models. For each of the models, we confirm that, if one in-

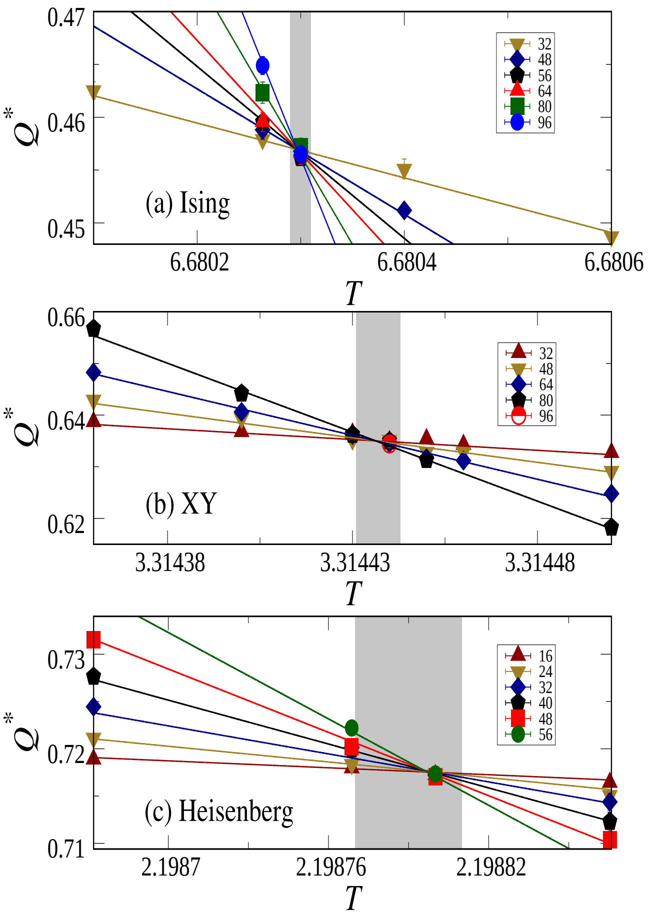

Figure 2. The Binder cumulant Q with finite-size corrections being subtracted, namely $Q^*(L,T) = Q(L,T) - b(\ln L)^{-\frac{1}{2}}$, with $b \approx$ 0.0991, 0.1069, and 0.1160 according to the preferred least-squares fits for the Ising (a), XY (b), and Heisenberg (c) models, respectively. The solid lines are drawn according to the fits, and the shadows mark T_c and their error margins. The plot for the XY case has appeared in the main text and we hereby include it for completeness.

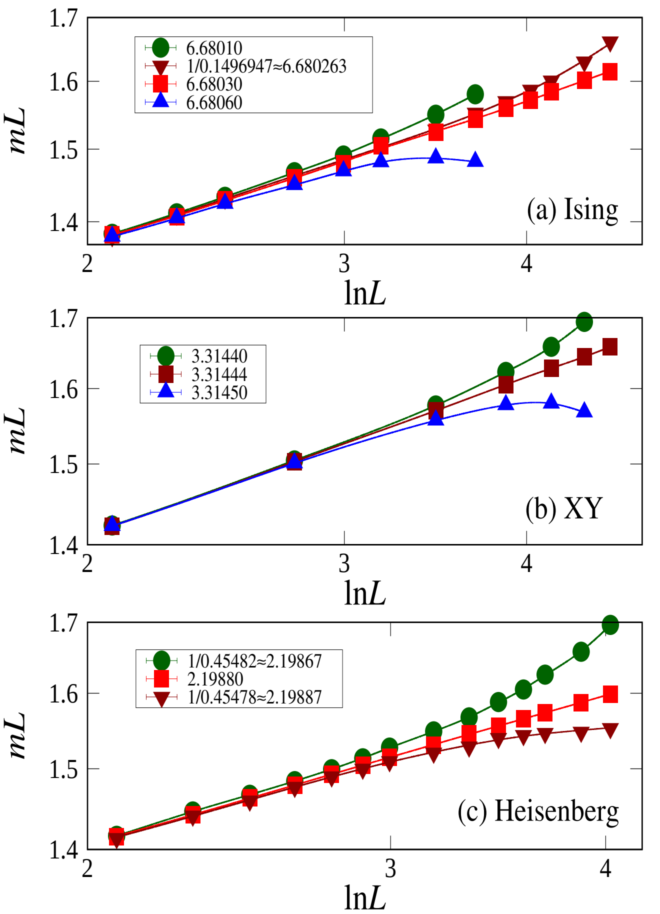

Figure 3. The magnetization density m rescaled by its mean-field factor $L^{y_h - d}$ ($y_h = 3$, $d = 4$) versus $\ln L$ around $T_c = 6.680\,30$, $3.314\,44$, and $2.198\,80$ in a log-log scale, for the Ising (a), XY (b), and Heisenberg (c) models, respectively. For each of the models, linearity is observed at T_c, and the bending-up or -down feature with respect to the linearity indicates the deviation from criticality. The plot for the XY case has appeared in the main text and we hereby include it for completeness.

cludes both q_1 and q_2 terms and let $\hat{p} = 1/2$ and $2y_h - d = 2$ fixed, stable fits with $\chi^2/\mathrm{DF} \lessapprox 1$ can be achieved. If the mean-field exponent $2y_h - d$ is free in the fits, we obtain $2y_h - d \approx 2.00$ for each model, in perfect agreement with the exact value 2 within error bars. For the Ising and XY models (for which we have Monte Carlo data with $L_{\max} = 96$), we achieve stable fits with $\hat{p}$ being free, which yield $\hat{p} \approx 0.5$, consistent with the prediction $\hat{p} = 1/2$.

FSS OF THE TWO-POINT CORRELATION $g(L/2, L)$

We perform FSS for the large-distance plateau of two-point correlation $g(r, L)$, by fitting the finite-size $g(L/2, L)$ data to the ansatz

$$g(L/2, L) = v_1 L^{2y_h - 2d} (\ln L)^{\hat{p}} + v_2 L^{2y_h - 2d} \quad (3)$$

for the critical XY model. We perform fits under the situations that $2y_h - 2d = -2$ and $\hat{p} = 1/2$ are both fixed, and that only one of them is fixed. The fits are summarized by Table V, which demonstrates that the inclusion of both v_1 and v_2 terms correctly produces the mean-field exponent y_h. As an instance, one of the fits yields $2y_h - 2d = -1.99(1)$ with $L_{\min} = 24$ and $\chi^2/\mathrm{DF} \approx 0.8$, which is in good agreement with the exact $2y_h - 2d = -2$. By comparing the fits with $\hat{p} = 1/2$ being fixed, we estimate $y_h = 3.01(2)$. As a further verification, once the mean-field exponent $2y_h - 2d = -2$ is fixed, the estimate $\hat{p} = 0.5(1)$ is again consistent with the prediction $\hat{p} = 1/2$.

* jplv2014@ahnu.edu.cn
† chenkun0228@gmail.com
‡ yjdeng@ustc.edu.cn

[1] R. Kenna, "Universal scaling relations for logarithmic-correction exponents," Chapter 1, Order, Disorder and Criticality, Advanced Problems of Phase Transition Theory, Vol. 3, Y. Holovatch ed (World Scientific, 2012).
[2] E. Luijten, *Interaction range, universality and the upper critical dimension* (Delft University Press, 1997).
[3] M.-H. Hu and J.-P. Lv, unpublished results (2019).

Table I. Fits of the finite-size Monte Carlo data of the Binder cumulant Q to (1) for the Ising ($n = 1$) model. The missing entries mean that the corresponding high-order corrections are not included or that the fits are performed right at $T_c = 6.680\,30$.

$L_{\min}$	χ^2/DF	T_c	$\hat{y}_t$	$\hat{p}$	Q_c	a	b	c
8	61.6/45	6.680 310(6)	$-0.3(2)$	0.5(1)	0.44(2)	0.04(1)	0.13(1)	
32	9.0/16	6.680 307(9)	$-0.5(5)$	0.6(1)	0.456947	0.05(3)	0.12(1)	
32	10.7/17	6.680 302(6)	$1/6$	0.59(9)	0.456947	0.0205(8)	0.11(1)	
40	9.2/12	6.680 302(9)	$1/6$	0.6(2)	0.456947	0.020(1)	0.11(3)	
24	34.1/22	6.680 292(2)	$1/6$	0.5	0.456947	0.0207(8)	0.1004(3)	
32	11.6/18	6.680 297(3)	$1/6$	0.5	0.456947	0.0206(8)	0.0991(5)	
40	9.4/13	6.680 298(3)	$1/6$	0.5	0.456947	0.020(1)	0.0987(6)	
48	2.0/8	6.680 301(4)	$1/6$	0.5	0.456947	0.019(1)	0.098(1)	
56	1.7/5	6.680 302(5)	$1/6$	0.5	0.456947	0.019(2)	0.098(1)	
24	18.8/22	6.680 307(4)	$1/6$	0.5	0.456947	0.0203(8)	0.17(1)	-0.11(2)
32	10.7/17	6.680 301(5)	$1/6$	0.5	0.456947	0.0205(8)	0.13(3)	-0.05(5)
40	9.2/12	6.680 302(8)	$1/6$	0.5	0.456947	0.020(1)	0.13(7)	0.0(1)
12	14.8/8			0.5	0.441(2)		0.130(3)	
16	6.2/7			0.5	0.446(3)		0.119(5)	
20	2.8/6			0.5	0.451(4)		0.110(7)	
32	1.8/5			0.5	0.457(7)		0.10(1)	
40	1.2/4			0.5	0.460(8)		0.09(2)	
8	10.1/9			0.5	0.460(5)		0.137(2)	-0.06(2)
20	2.6/6			0.56(3)	0.456947		0.107(5)	
32	1.8/5			0.50(7)	0.456947		0.099(9)	
8	10.1/9			0.48(4)	0.456947		0.140(6)	-0.07(2)

Table II. Fits of the finite-size Monte Carlo data of the Binder cumulant Q to (1) for the XY ($n = 2$) model. The missing entries mean that the corresponding high-order corrections are not included or that the fits are performed right at $T_c = 3.314\,44$.

$L_{\min}$	χ^2/DF	T_c	$\hat{y}_t$	$\hat{p}$	Q_c	a	b	c
8	29.9/35	3.314 440(3)	$-0.2(3)$	0.5(1)	0.62(3)	0.05(2)	0.13(2)	
8	30.6/36	3.314 440(3)	$1/10$	0.5(1)	0.62(3)	0.0360(8)	0.13(2)	
8	30.7/37	3.314 439(2)	$1/10$	0.5	0.63(1)	0.0360(8)	0.126(2)	
16	25.8/30	3.314 439(2)	$1/10$	0.5	0.62(1)	0.0360(8)	0.126(4)	
32	23.6/23	3.314 442(4)	$1/10$	0.5	0.62(1)	0.0360(8)	0.13(1)	
16	50.4/31	3.314 430(1)	$1/10$	0.5	0.635	0.0358(8)	0.1091(2)	
32	26.9/24	3.314 434(1)	$1/10$	0.5	0.635	0.0359(8)	0.1078(3)	
48	18.1/17	3.314 436(2)	$1/10$	0.5	0.635	0.0360(8)	0.1069(7)	
16	26.4/30	3.314 437(2)	$1/10$	0.5	0.635	0.0360(8)	0.131(4)	-0.035(7)
32	23.7/23	3.314 440(4)	$1/10$	0.5	0.635	0.0360(8)	0.15(3)	-0.07(4)
8	1.9/5			0.5	0.63(1)		0.123(2)	
16	1.9/4			0.5	0.63(1)		0.123(4)	
32	0.8/3			0.5	0.63(1)		0.11(1)	
8	1.9/4			0.5(2)	0.63(2)		0.12(2)	
32	0.8/3			0.53(5)	0.635		0.111(7)	
48	0.6/2			0.6(1)	0.635		0.12(2)	
8	1.8/4			0.59(9)	0.635		0.114(8)	0.01(3)
16	1.0/3			0.4(1)	0.635		0.15(4)	-0.07(9)

Table III. Fits of the finite-size Monte Carlo data of the Binder cumulant Q to (1) for the Heisenberg ($n = 3$) model. The missing entries mean that the corresponding high-order corrections are not included or that the fits are performed right at $T_c = 2.198\,80$.

$L_{\min}$	χ^2/DF	T_c	$\hat{y}_t$	$\hat{p}$	Q_c	a	b	c
24	22.4/24	2.198 796(8)	1/22	0.59(7)	0.728	0.045(1)	0.108(9)	
12	41.0/44	2.198 791(3)	1/22	0.5	0.72(1)	0.045(1)	0.108(2)	
16	32.7/36	2.198 796(4)	1/22	0.5	0.72(1)	0.046(1)	0.114(5)	
20	25.4/28	2.198 799(6)	1/22	0.5	0.72(1)	0.046(1)	0.118(8)	
24	22.4/24	2.198 797(8)	1/22	0.5	0.72(1)	0.045(1)	0.11(1)	
20	31.2/29	2.198 785(2)	1/22	0.5	0.728	0.045(1)	0.0981(2)	
24	23.9/25	2.198 788(2)	1/22	0.5	0.728	0.045(1)	0.0976(3)	
32	20.9/17	2.198 789(3)	1/22	0.5	0.728	0.045(1)	0.0974(5)	
12	42.3/44	2.198 788(3)	1/22	0.5	0.728	0.045(1)	0.108(2)	-0.015(4)
16	33.5/36	2.198 793(4)	1/22	0.5	0.728	0.046(1)	0.116(5)	-0.028(9)
20	25.6/28	2.198 795(5)	1/22	0.5	0.728	0.046(1)	0.12(1)	-0.04(2)
24	22.6/24	2.198 795(7)	1/22	0.5	0.728	0.045(1)	0.12(2)	-0.03(3)
16	8.0/8			0.5	0.72(1)		0.120(3)	
20	2.6/6			0.5	0.71(1)		0.121(4)	
24	2.5/5			0.5	0.71(1)		0.122(6)	
32	0.9/3			0.5	0.71(1)		0.12(1)	
12	10.4/9			0.5	0.70(1)		0.111(4)	0.04(3)
16	6.8/7			0.5	0.70(1)		0.11(1)	0.06(5)
20	2.3/5			0.5	0.70(2)		0.11(2)	0.1(1)
24	2.3/4			0.5	0.70(4)		0.10(5)	0.1(2)

Table IV. Fits of the finite-size Monte Carlo data of the magnetic susceptibility χ_0 to (2) for the critical Ising ($n = 1$), XY ($n = 2$) and Heisenberg ($n = 3$) models.

n	$L_{\min}$	χ^2/DF	$2y_h - d$	$\hat{p}$	q_1	q_2
1	8	6.3/9	2	0.56(4)	1.2(1)	0.7(2)
	10	5.0/8	2	0.50(7)	1.4(3)	0.5(3)
	12	1.3/7	2	0.6(1)	1.0(3)	1.0(3)
	8	8.2/10	2	0.5	1.440(6)	0.46(1)
	10	5.0/9	2	0.5	1.448(8)	0.45(1)
	12	3.4/8	2	0.5	1.44(1)	0.46(2)
	16	2.7/7	2	0.5	1.45(1)	0.45(3)
	20	0.8/6	2	0.5	1.47(2)	0.40(4)
	8	6.2/9	2.006(5)	0.5	1.35(6)	0.56(7)
	10	5.0/8	2.001(7)	0.5	1.4(1)	0.5(1)
	12	1.3/7	2.01(1)	0.5	1.2(1)	0.7(2)
	16	1.1/6	2.02(1)	0.5	1.2(2)	0.8(3)
	20	0.6/5	2.01(2)	0.5	1.3(3)	0.6(4)
2	8	0.6/4	2	0.45(4)	1.5(2)	0.3(2)
	16	0.2/3	2	0.4(1)	2(1)	0(1)
	8	1.8/5	2	0.5	1.254(6)	0.49(1)
	16	1.1/4	2	0.5	1.25(1)	0.50(2)
	32	0.4/3	2	0.5	1.23(2)	0.54(5)
	48	0.1/2	2	0.5	1.21(4)	0.58(9)
	8	0.5/4	1.995(4)	0.5	1.32(6)	0.41(7)
	16	0.2/3	1.99(1)	0.5	1.4(2)	0.3(2)
3	10	26.1/11	2	0.5	1.109(3)	0.565(5)
	12	13.4/10	2	0.5	1.100(4)	0.581(7)
	16	8.1/8	2	0.5	1.089(6)	0.60(1)
	20	4.0/6	2	0.5	1.075(9)	0.63(2)
	24	4.0/5	2	0.5	1.07(1)	0.63(3)
	28	0.5/4	2	0.5	1.05(2)	0.68(4)
	32	0.4/3	2	0.5	1.06(3)	0.66(5)
	16	3.3/7	1.98(1)	0.5	1.4(1)	0.2(2)
	20	2.9/5	1.98(2)	0.5	1.3(2)	0.3(3)

Table V. Fits of the finite-size Monte Carlo data of the two-point correlation $g(L/2, L)$ to (3) for the critical XY model.

$L_{\min}$	χ^2/DF	$2y_h - 2d$	$\hat{p}$	v_1	v_2
24	4.0/5	-2	0.5	0.607(3)	0.339(6)
32	2.2/4	-2	0.5	0.613(6)	0.33(1)
40	1.5/3	-2	0.5	0.61(1)	0.34(2)
24	3.3/4	$-1.99(1)$	0.5	0.54(8)	0.42(9)
32	1.3/3	$-2.02(2)$	0.5	0.8(2)	0.1(3)
40	1.3/2	$-2.02(4)$	0.5	0.8(4)	0.1(6)
16	28.5/5	-2	0.52(5)	0.55(8)	0.41(9)
24	4.8/4	-2	0.5(1)	0.7(3)	0.2(3)
